# Mechanism of Gut Microbiota Dysbiosis in Insulin Resistance Polycystic Ovary Syndrome

**DOI:** 10.2174/0118715303429583260303181915

**Published:** 2026-04-13

**Authors:** Fatimah Usman, Masagus Irsan Saleh, Kemas Yusuf Effendi, Peby Maulina Lestari, Widjiati Widjiati

**Affiliations:** 1 Doctoral Program of Biomedic Science, Faculty of Medicine, Sriwijaya University, Palembang, Indonesia;; 2 Department of Pharmacology, Sriwijaya University, Palembang, Indonesia;; 3 Division of Reproductive Endocrinology and Infertility, Department of Obstetrics and Gynecology, Dr. Mohammad Hoesin General Hospital, Palembang, Indonesia;; 4 Division of Maternal-Fetal Medicine, Department of Obstetrics and Gynecology, Dr. Mohammad Hoesin General Hospital, Palembang, Indonesia;; 5 Department of Embryology, Faculty of Veterinary Medicine, University of Airlangga, Surabaya, Indonesia

**Keywords:** PCOS, dysbiosis, gut microbiota, Zonulin, SCFAs, IGF-1, HOMA-IR, ovarian follicle, insulin resistance

## Abstract

**Introduction:**

Polycystic Ovary Syndrome (PCOS) is a complex endocrine disorder associated with insulin resistance, hyperandrogenism, and infertility. Gut microbiota dysbiosis is suspected to play a significant role in the pathogenesis of PCOS through the gut-brain-gonad axis, an axis that remains incompletely understood.

**Methods:**

This study aims to evaluate the role of gut microbiota dysbiosis in the mechanism of PCOS with insulin resistance by analyzing the abundance of *Lactobacillus *sp., levels of Zonulin, Short Chain Fatty Acids (SCFAs), Insulin-Like Growth Factor-1 (IGF-1), and ovarian follicle count in a rat model of PCOS with insuline resistance, which in this study devided into 2 groups, control and treatment group, consist of 20 rats each. This experimental study employed an SEM- PLS approach to assess the relationships among microbiota, inflammation, metabolism, and ovarian function variables in an insulin-resistant PCOS rat model with insulin resistance.

**Results:**

The results showed decreases in *Lactobacillus *sp., SCFAs, and ovarian follicle counts, along with an increase in IGF-1 levels in PCOS model rats.

**Discussion:**

The study, utilizing an insulin-resistant Polycystic Ovary Syndrome (PCOS) rat model, established a strong, statistically significant link between gut microbiota dysbiosis and endocrine/metabolic dysfunction. These were observed through increased levels of key markers: Zonulin (Control: 3.775, Treatment: 5.545), HOMA-IR (Control: 78.3400, Treatment: 140.9760), and IGF-1 (Control: 141.9665, Treatment: 189.5700). The Structural Equation Modeling (SEM) analysis confirmed two primary pathogenic pathways: (1) Gut Microbe/Metabolite Pathway: A reduction in beneficial *Lactobacillus *sp. was linked to lower SCFA levels, which influenced IGF-1 and negatively affected ovarian follicle development; (2) Inflammation/Insulin Resistance Pathway: The model established a statistically significant cascade where PCOS induction was strongly associated with Zonulin (β = -0.717), which then positively predicted HOMA-IR (β = 0.630), and HOMA-IR, in turn, strongly predicted IGF-1 (β = 0.662), defining the mechanism as PCOS → Zonulin → HOMA-IR → IGF-1.

**Conclusion:**

The dysbiosis mechanism involving gut microbiota in this study’s model includes PCOS, Zonulin, HOMA-IR, and IGF-1, as these variables demonstrated statistically significant relationships

## 1 INTRODUCTION

Polycystic ovary syndrome (PCOS) is a complex endocrine disorder affecting approximately 4–18% of women of reproductive age worldwide [1]. This condition is characterized by oligo/anovulation, hyperandrogenism, and polycystic ovarian morphology, and is often associated with insulin resistance and various metabolic complications such as obesity, type 2 diabetes mellitus, and cardiovascular disease [1]. The primary complication of PCOS is infertility, affecting 5–10% of patients and significantly impacting quality of life [2]. Despite extensive research, the pathophysiology of PCOS remains incompletely understood, necessitating further exploration of more comprehensive biological mechanisms.

In recent years, scientific attention has increasingly focused on the role of the gut microbiota in regulating systemic health. The bidirectional signaling pathway known as the gut-brain-gonad axis plays a crucial role in maintaining metabolic, hormonal, and immunological homeostasis [3]. Gut microbiota dysbiosis, defined as an imbalance in the composition of intestinal microorganisms, has been linked to chronic metabolic disturbances such as obesity, insulin resistance, and systemic inflammation—all of which are hallmark features of PCOS [4]. The microbiota produces metabolites such as short-chain fatty acids (SCFAs), which influence gut permeability, immune activation, and even ovarian function through neuroendocrine signaling [5].

Several studies have confirmed an association between gut dysbiosis and PCOS, including alterations in the *Lactobacillus* genus, elevated levels of zonulin (a marker of leaky gut), and disrupted expression of SCFAs and growth-related hormones such as IGF-1 [6-8]. However, most of these studies remain correlational and have yet to systematically integrate the gut-brain-gonad axis in explaining the pathogenic mechanisms of PCOS, particularly through experimental approaches using animal models that reflect insulin resistance. Moreover, the functional relationship between intestinal biomarkers and ovarian follicle count—a key clinical feature of PCOS—has not been thoroughly investigated.

This study aims to address these gaps by examining the role of the gut microbiota in the gut-brain-gonad axis using an insulin-resistant rat model of PCOS. The primary focus is on analyzing changes in zonulin, IGF-1, and HOMA-IR levels. This integrative approach enables a deeper understanding of the systemic mechanisms linking gut microbiota dysbiosis with endocrine and reproductive dysfunctions.

Various studies have shown that a reduction in *Lactobacillus* species correlates with increased intestinal permeability, mediated by the protein zonulin [9]. Zonulin plays a role in loosening intestinal epithelial tight junctions, and its levels are elevated in several inflammatory and metabolic conditions, including PCOS [9]. This increased permeability facilitates the translocation of lipopolysaccharides (LPS) into systemic circulation, which can trigger immune activation and insulin resistance [10].

On the other hand, elevated IGF-1 levels, influenced by gut-brain axis signaling, have been shown to contribute to granulosa cell proliferation and androgen production, thereby worsening ovarian dysfunction [11, 12]. Insulin resistance, commonly assessed using the HOMA-IR index, remains a key translational research parameter. In the context of PCOS, insulin resistance not only exacerbates hyperandrogenism but also impairs ovulation and reduces the number of developing ovarian follicles [13]. Follicle count is a critical diagnostic criterion for PCOS and reflects disturbances in oocyte maturation [14]. Targeting gut microbiota may indirectly modulate these parameters by improving systemic metabolic and hormonal profiles [14].

The gut microbiota is a complex community of microorganisms that plays a crucial role in maintaining metabolic, immune, and hormonal homeostasis [15]. Dysbiosis, or imbalance in microbial composition, can disrupt intestinal barrier integrity by altering tight junction proteins such as ZO-1 and occludin, leading to leaky gut and LPS translocation into systemic circulation [16]. LPS induces chronic inflammation and insulin resistance—two central elements in the pathogenesis of polycystic ovary syndrome [4]. Additionally, the microbiota modulates adaptive immune responses by regulating cytokine profiles, such as the protective IL-10 response induced by *Lactobacillus* and the pro-inflammatory IL-17 response driven by *Prevotella*, which worsens ovarian inflammation [17]. Thus, the gut microbiota plays a multifaceted role in immune-metabolic regulation and hormonal balance, making it a promising therapeutic target for PCOS management.

## 2 MATERIALS AND METHODS

This study is a true experimental study employing a post-test control-only group design, aimed at evaluating the impact of gut microbiota disruption on the gut–brain–gonad axis in an insulin-resistant PCOS animal model. This study was held from December 1^st^ to 28^th^, 2024, including adaptation and treatment periods in the Animal Laboratory, Faculty of Veterinary Medicine, Universitas Airlangga, Surabaya, Indonesia. This study used female Wistar strain white rats, aged 3 months, weighing 130–160 g, healthy, eating well, and actively moving, with no prior use in other studies. All of the rats were obtained from Laboratory of Faculty of Veterinary Medicine, Universitas Airlangga. Two groups were used without blinding: a control group (healthy rats) and a treatment group (rats induced with PCOS using testosterone propionate). Observed variables included intestinal permeability (Zonulin) and metabolic indicators (IGF-1 and HOMA-IR). A total of 40 female Wistar rats were randomly divided into two groups of 20 rats each. The sample size was calculated utilizing Cohen’s d formula. Researchers were aware of this group allocation.

PCOS induction was carried out by oral administration of a testosterone propionate dose of 100 mg/kgBW/day for 21 days. Following treatment and recovery, blood, ovary, and colon samples were collected. Insulin resistance was validated by checking HOMA-IR (fasting insulin (U/L)) x (fasting glucose (mmol/L)) / 22,5. IGF-1 levels were analyzed using ELISA, Zonulin expression was examined *via* immunohistochemistry, and HOMA-IR values were calculated by multiplying fasting insulin by fasting glucose levels. The data captured microbiological, immunological, metabolic, and endocrine dimensions involved in reproductive dysfunction caused by gut dysbiosis.

Data analysis was conducted using IBM SPSS Statistic Application utilizing the Structural Equation Modeling–Partial Least Squares (SEM-PLS) approach to evaluate causal relationships among the variables within the framework of the gut–brain–gonad axis. The units used in this research are zonulin (ng/mL), HOMA-IR (units), and IGF-1 (ng/mL).

## 3 RESULTS

Based on the results of the study, data normality for each variable was tested using the Shapiro–Wilk test (n = 20). A variable was considered normally distributed if *p* > 0.05. If both groups were normally distributed, an independent *t*-test was used; if one or both groups were not normally distributed, the Mann–Whitney *U* test was applied instead.

Based on Table **1**, the Zonulin variable was not normally distributed in the treatment group; therefore, it was analyzed using the Mann–Whitney *U* test, which yielded a *p*-value < 0.05, indicating a significant difference between groups. Meanwhile, HOMA-IR and IGF-1 variables were normally distributed and analyzed using the independent *t*-test, which also revealed significant differences between the control and treatment groups.

Descriptive mean analysis from the bivariate test showed that the average Zonulin level in the control group was 3.775, compared to 5.545 in the treatment group. This finding indicates a significant increase in Zonulin levels in the treatment group, as shown in the box plot (Fig. **1**).

Descriptive mean analysis from the bivariate test showed that the average HOMA-IR level in the control group was 78.3400, while in the treatment group it was 140.9760. This finding indicates a significant increase in HOMA-IR levels in the treatment group, as shown in the box plot (Fig. **2**).

Descriptive mean analysis from the bivariate test showed that the average IGF-1 level in the control group was 141.9665, while in the treatment group it was 189.5700. This finding indicates a significant increase in IGF-1 levels in the treatment group, as shown in the box plot (Fig. **3**).

The coefficient of determination (R^2^) measures the extent to which exogenous variables explain the variance in endogenous variables, with values ranging from 0 to 1. R^2^ is classified as substantial/very strong (0.76–0.99), strong (0.51–0.75), moderate (0.26–0.50), and very weak (0.00–0.25), where values closer to 1 indicate higher or near-perfect predictability of the independent variable toward the dependent variable (Zhang *et al*., 2023) [17].

Table **2** shows the structural path coefficients, indicating that HOMA-IR strongly predicts IGF-1 (β = 0.662), IGF-1 positively predicts ovarian follicles (β = 0.555), PCOS negatively predicts zonulin (β = –0.717), and zonulin positively predicts HOMA-IR (β = 0.630).

Table ****3**** shows the bootstrapping results, demonstrating that all structural paths are statistically significant, with high t-statistics (6.062–9.502) and *p*-values < 0.001, confirming the strength and stability of the estimated coefficients.

Table ****4**** presents the f ^2^ effect size values, demonstrating the magnitude of each predictor’s contribution within the structural model. All variables exhibit large effect sizes according to Cohen’s criteria, indicating strong explanatory power in their respective pathways. HOMA-IR exhibits a substantial influence on IGF-1 (f ^2^ = 0.78), while IGF-1 strongly affects ovarian follicle outcomes (f ^2^ = 0.45). Zonulin also shows a strong effect on HOMA-IR (f ^2^ = 0.66), supporting the link between increased intestinal permeability and insulin resistance. The largest effect is observed in the pathway from PCOS to Zonulin (f ^2^ = 1.06), suggesting that PCOS exerts a dominant influence on intestinal permeability markers.

Table **5** shows the Fornell–Larcker values, in which the square root of the AVE for each construct exceeds its correlations with other constructs, indicating satisfactory convergent and discriminant validity.

Table **6** shows the cross-loadings, indicating that each indicator loads highest on its corresponding construct relative to other constructs, providing additional support for discriminant validity.

Table **7** shows the HTMT ratios, all of which fall below the recommended threshold of 0.90, confirming adequate discriminant validity among the latent constructs.

Table ****8**** shows the blindfolding results, showing positive Q^2^ values (0.286–0.495) for all endogenous constructs, indicating that the model has meaningful predictive relevance.

Based on Table **9**, the adjusted R^2^ value for Zonulin is 0.511, indicating that Zonulin explains 51.1% of the variation in the endogenous variable of the study. The adjusted R^2^ values for IGF-1 and HOMA-IR are 0.423 and 0.382, respectively, meaning that the exogenous variables IGF-1 and HOMA-IR explain 42.3% and 38.2% of the variation in the endogenous variable.

Predictive relevance Q-Square (Q^2^) is used to evaluate the predictive capability of a model. A Q^2^ value greater than 0 indicates good predictive relevance, while a value less than 0 indicates poor fit. Q^2^ is calculated using SSE (sum of squared errors) and SSO (sum of squares observed), where SSE reflects prediction error, and SSO represents the total data variance; the smaller the SSE, the better the model’s predictive ability (Fig. **4**).

Based on Table **10**, all endogenous variables in the model, HOMA-IR, IGF-1, and Zonulin have positive Q^2^ values. This indicates that the model has adequate predictive power and can explain the observed variations in a relevant manner.

Table ****11**** shows Spearman correlation coefficients among the key variables examined in the study, revealing strong, consistent associations across the dataset. PCOS shows a strong negative correlation with *Lactobacillus * sp. (r = –0.915) and with ovarian follicles (r = –0.929), indicating that PCOS severity is associated with reduced beneficial microbiota and impaired follicular development. On the other hand, *Lactobacillus* sp. demonstrates strong positive correlations with ovarian follicles (r = 0.919) and moderate correlations with IGF-1 (r = 0.518), highlighting its potential protective metabolic and reproductive roles. Zonulin correlates positively with HOMA-IR (r = 0.630) and negatively with *Lactobacillus *sp. (r = –0.644), supporting the connection between intestinal permeability, dysbiosis, and metabolic imbalance. HOMA-IR exhibits strong correlations with IGF-1 (r = 0.662) and ovarian follicles (r = 0.828), reinforcing the central role of insulin resistance in driving endocrine and reproductive dysfunction. Overall, the correlation matrix underscores a coherent pathway in which PCOS-induced dysbiosis increases Zonulin and insulin resistance, elevates IGF-1, and ultimately disrupts ovarian follicle development.

The CI results in Table **12** indicate that both estimated path coefficients are statistically robust (each 95% interval does not cross zero), confirming the significance of the relationships. HOMA-IR shows a positive effect on both Follicle Count (O = 0.37; CI 0.17–0.56) and IGF-1 (O = 0.66; CI 0.48–0.80), while IGF-1 also shows a strong positive effect on Follicle Count (O = 0.56; CI 0.35–0.71). In contrast, PCOS consistently exhibits negative effects with Follicle Count, HOMA-IR, IGF-1, and Zonulin, with relatively narrow confidence intervals, indicating reliable estimates. Zonulin shows positive effects on Follicle Count, HOMA-IR, and IGF-1, with all intervals remaining above zero, demonstrating consistent and meaningful contributions.

Based on Table **13**, all relationships in the model are statistically significant, with T-statistics greater than 1.96 and *p*-values less than 0.05, indicating that all direct effects tested in the model are statistically significant. This strengthens the model's reliability in explaining the associations among the variables involved in the pathophysiological pathway.

Based on Table **14**, the statistical analysis results show that the pathway PCOS → Zonulin → HOMA-IR → IGF-1 is significant. A T-statistic value of 3.682 (> 1.96) and a *p*- value of 0.000 (< 0.05) indicate that the relationships in this pathway are statistically meaningful. This suggests that the dysbiosis mechanism in the insulin-resistant PCOS rat model involves Zonulin, HOMA-IR, and IGF-1.

Additional data are available in the supplementary materials, including bivariate and normality tests for Zonulin, IGF-1, and HOMA IR.

## 4 DISCUSSION

The current investigation demonstrated that Zonulin levels were significantly elevated in the insulin-resistant Polycystic Ovary Syndrome (PCOS) rat model. Zonulin is a key regulatory protein that governs intestinal permeability by modulating epithelial tight junctions. This mechanism is crucial for preserving the equilibrium between optimal nutrient absorption and defense against luminal pathogens. However, in pathological states, including chronic inflammation, obesity, and insulin resistance, Zonulin expression is typically augmented, as observed in this study. This elevated Zonulin expression promotes compromised gut barrier function, or “gut leakage”, facilitating the translocation of lipopolysaccharides (LPS) derived from Gram-negative bacteria into the systemic circulation. Systemic LPS subsequently activates Toll-like receptor 4 (TLR4) on immune and metabolic cells, initiating a cascade of systemic inflammation characterized by the heightened release of pro-inflammatory cytokines such as TNF-α and IL-6. This inflammatory milieu is known to exacerbate insulin signaling disruption and contribute to the maintenance of insulin resistance in PCOS. These observations align with existing literature correlating elevated Zonulin concentrations with insulin resistance in metabolic syndrome, type 2 diabetes, and experimental PCOS models exhibiting compromised gut permeability, microbial dysregulation, and metabolic inflammation.

The observation of increased Insulin-like Growth Factor-1 (IGF-1) levels in the insulin-resistant PCOS rat model is attributed to compensatory hyperinsulinemia, which stimulates the excessive synthesis of both IGF-1 and androgens. This resultant hormonal imbalance precipitates ovulatory dysfunction *via* abnormal folliculogenesis. Furthermore, gut microbiota dysbiosis exacerbates this pathology by increasing intestinal permeability and systemic inflammation through activation of the NF-κB pathway and subsequent release of pro-inflammatory cytokines (IL-6, TNF-α). This process further compromises insulin sensitivity and augments IGF-1 levels. Notably, elevated IGF-1 concentrations also contribute to systemic metabolic derangements, encompassing abdominal obesity, dyslipidemia, and impaired glucose tolerance, thereby escalating the risk for type 2 diabetes mellitus and cardiovascular diseases. This established pathological cycle intensifies both the metabolic and reproductive dysfunctions characteristic of PCOS, underscoring that therapeutic interventions aimed at regulating IGF-1, enhancing insulin sensitivity, and modulating the gut microbiota hold significant promise as viable treatment strategies.

The elevated Homeostasis Model Assessment of Insulin Resistance (HOMA-IR) levels observed in the insulin-resistant PCOS rat model reflect impaired insulin homeostasis, rooted in reduced cellular insulin responsiveness, which, in turn, provokes compensatory hyperinsulinemia. Insulin resistance is significantly influenced by hormonal factors, particularly hyperandrogenism, and by systemic inflammation, mediated through NF-κB pathway activation and the induction of pro-inflammatory cytokines, including IL-6 and TNF-α. Supporting evidence from previous studies, such as Rusnasari's finding of insulin resistance in 82.9% of PCOS cases and DeUgarte's reported prevalence of 64.4% following BMI adjustment, reinforces the critical role of these underlying mechanisms. Moreover, a demonstrated positive correlation between HOMA-IR and body mass index (BMI), coupled with the proven efficacy of interventions such as Metformin and Myoinositol in reducing HOMA-IR, underscores the clinical imperative of managing insulin resistance in this population. The reliability of HOMA-IR as a surrogate measure of insulin resistance is further validated by its correlation with the Insulin Resistance Index (IRI) (r = 0.5–0.57) and its utility in high-fat diet animal models. Pathophysiologically, elevated HOMA-IR levels confer increased susceptibility to type 2 diabetes mellitus and cardiovascular disease, thereby establishing insulin resistance as a central determinant in PCOS pathogenesis and a key therapeutic target. Overall, this study decisively demonstrates a significant association between alterations in Zonulin, HOMA-IR, and IGF-1 levels and the dysbiotic mechanisms implicated in the PCOS rat model.

The interrelationship between gut microbiota dysbiosis and systemic endocrine disruption extends to circadian and stress-response axes. Emerging evidence suggests that the gut microbiome is intrinsically linked to systemic circadian processes, notably through the protective influence of pineal melatonin, which mitigates gut permeability and dysbiosis, thereby opposing the catabolic effects of cortisol [18]. Furthermore, butyrate, a critical Short-Chain Fatty Acid (SCFA) metabolite of the microbiota, has been demonstrated to augment melatonin synthesis within intestinal epithelial cells. Analogous to melatonin itself, butyrate exerts a suppressive effect on the nuclear translocation of the glucocorticoid receptor (GR)-alpha [19]. Consequently, the observed changes in the gut microbiota in this study may be closely associated with alterations in both local/circadian melatonin and circadian/stress-related cortisol levels, providing a more comprehensive understanding of the complex pathophysiology of PCOS. This conceptual framework suggests that modulating the gut-brain-gonad axis, particularly by rebalancing the melatonin-cortisol signaling dynamics through targeted manipulation of the gut microbiota, represents a promising avenue for novel PCOS therapeutic strategies.

While this study establishes a strong, statistically significant mechanism within the rat model, the interpretation must account for potential confounding factors and alternative pathways. Differences in dietary intake (*e.g*., high-fat, high- sugar diets) are known to critically and independently modulate the gut microbiota and to influence both intestinal barrier function markers, like Zonulin, and metabolic outcomes, thus potentially confounding the direct causal links observed. Furthermore, compromised gut integrity leads to systemic exposure to Lipopolysaccharide (LPS). While Zonulin initiates the “gut leakage”, LPS can induce insulin resistance through mechanisms that are parallel or partially independent of Zonulin levels, primarily by activating Toll-like receptor 4 (TLR4) on immune and metabolic tissues, which subsequently initiates systemic inflammation (*via* TNF-α and IL-6) that disrupts insulin signaling. Acknowledging these alternative, LPS-mediated mechanisms ensures a comprehensive understanding of the complex pathogenesis of insulin resistance in PCOS.

The extrapolation of these mechanistic findings from the Wistar rat model to human PCOS pathophysiology requires a careful consideration of translational limitations. Significant physiological differences exist between rodents and humans, particularly in the complexity of the gut microbiome, ovarian regulatory pathways, and overall hormonal feedback systems. The use of the testosterone propionate induction model effectively mimics key features of PCOS like hyperandrogenism and insulin resistance, but it may not fully capture the genetic, environmental, and chronic inflammatory heterogeneity observed in human patients. Therefore, while the defined gut-brain-gonad axis pathway provides a valuable mechanistic framework for PCOS, the specific strength of the causal relationships (path coefficients) and the overall predictive relevance of this model may differ when applied to the human clinical setting. Future clinical research is imperative to validate the sequence and quantitative influence of Zonulin, HOMA-IR, and IGF-1 within the human PCOS population.

A primary limitation of this study is its reliance on an experimental animal model (Wistar rats induced with testosterone propionate). While effective for studying mechanistic pathways, the model cannot fully replicate the complex genetic, environmental, and heterogeneous pathophysiology observed in human PCOS. Furthermore, the study focused on a defined pathway (PCOS → Zonulin → HOMA-IR → IGF-1), which may overlook important confounding factors such as detailed dietary composition and parallel mechanisms, like LPS-mediated inflammation that drives insulin resistance independently of Zonulin regulation. The extrapolation of the derived path coefficients and predictive relevance to the human clinical setting, therefore, requires cautious translational validation.

## CONCLUSION

Based on the results obtained, several key findings were identified that contribute to a deeper understanding of the pathogenesis of PCOS and the mechanisms involving the interaction between gut microbiota and metabolic and endocrine systems: Zonulin levels increased in insulin-resistant PCOS model rats; Insulin-like Growth Factor-1 (IGF-1) levels increased in insulin-resistant PCOS model rats; Homeostasis Model Assessment of Insulin Resistance (HOMA-IR) levels increased in insulin-resistant PCOS model rats; Path analysis results showed that the dysbiosis mechanism involving gut microbiota in this study’s model includes Polycystic Ovary Syndrome, Zonulin, HOMA-IR, and Insulin-like Growth Factor-1, as these variables demonstrated statistically significant relationships.

## Figures and Tables

**Fig. (1):**
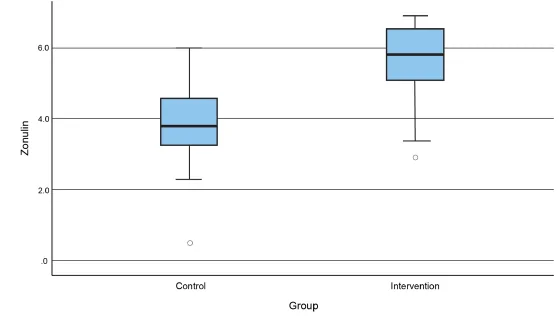
Box plot comparison of zonulin levels between PCOS-insulin resistance group and control group.

**Fig. (2):**
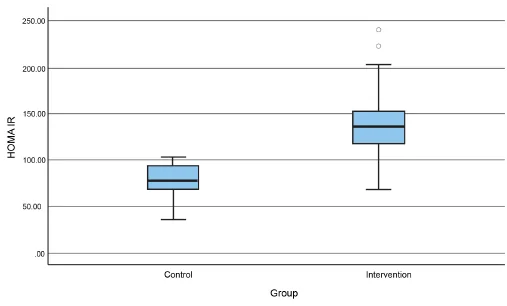
Box plot comparison of HOMA-IR levels between PCOS-insulin resistance group and control group.

**Fig. (3):**
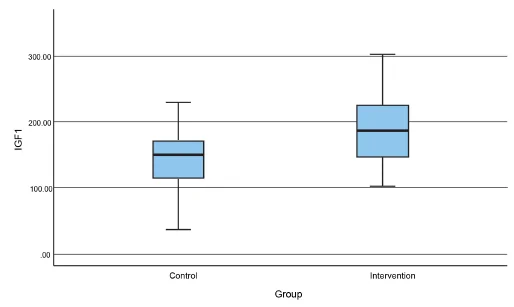
Box plot comparison of IGF-1 levels between PCOS-insulin resistance group and control group.

**Fig. (4):**
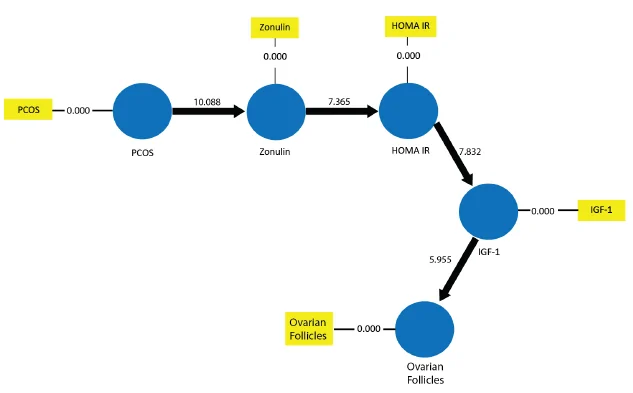
PLS-SEM structural path model showing the relationships among PCOS, Zonulin, HOMA-IR, IGF-1, and Follicle.

**Table 1:** Normality test and bivariate analysis of zonulin, HOMA-IR, and IGF-1 variables.

**Variable**	**Control**	**Shapiro-Wilk**	**Mann-Whitney Test**	**T-Test**
		Sig.	Sig.	Sig.
Zonulin	Control	0.413	0.000	-
	Dependent	0.027		
HOMA IR	Control	0.231	-	0.000
	Dependent	0.106		
IGF1	Control	0.895	-	0.004
	Dependent	0.589	-	-

**Table 2:** Path coefficient.

-	**Ovarium Follicle**	**HOMA IR**	**IGF1**	**PCOS**	**Zonulin**
**Ovarium Follicle**	-		-	-	-
**HOMA IR**	-	-	0.662	-	-
**IGF1**	0.555	-	-	-	-
**PCOS**	-	-	-	-	-0.717
**Zonulin**	-	0.63	-	-	-

**Table 3:** Bootstrapping path coefficient.

-	**Original Sample (O)**	**Sample Mean (M)**	**Standard Deviation (STDEV)**	**T Statistics (|O/STDEV|)**	***P* Values**
**HOMA IR → IGF1**	0.662	0.659	0.08	8.23	0
**IGF1 → Ovarian follicle**	0.555	0.552	0.092	6.062	0
**PCOS → Zonulin**	-0.717	-0.717	0.075	9.502	0
**Zonulin → HOMA IR**	0.63	0.629	0.088	7.155	0

**Table 4:** F^2^-test.

-	**Ovarian Follicles**	**HOMA IR**	**IGF1**	**PCOS**	**Zonulin**
**Ovarian Follicles**	-	-	-	-	-
**HOMA IR**	-	-	0.78	-	-
**IGF1**	0.45	-	-	-	-
**PCOS**	-	-	-	-	1.06
**Zonulin**	-	0.66	-	-	-

**Table 5:** Fornell-Larcker criterion.

-	**Ovarium Follicle**	**HOMA IR**	**IGF1**	**PCOS**	**Zonulin**
**Ovarium Follicle**	1	-	-	-	-
**HOMA IR**	0.828	1	-	-	-
**IGF1**	0.555	0.662	1	-	-
**PCOS**	-0.929	-0.858	-0.565	1	-
**Zonulin**	0.686	0.63	0.431	-0.717	1

**Table 6:** Cross loading.

-	**Ovarium Follicle**	**HOMA IR**	**IGF1**	**PCOS**	**Zonulin**
**Ovarium Follicle**	1	0.828	0.555	-0.929	0.686
**HOMA IR**	0.828	1	0.662	-0.858	0.63
**IGF1**	0.555	0.662	1	-0.565	0.431
**PCOS**	-0.929	-0.858	-0.565	1	-0.717
**Zonulin**	0.686	0.63	0.431	-0.717	1

**Table 7:** HTMT

-	**Ovarium Follicle**	**HOMA IR**	**IGF1**	**PCOS**	**Zonulin**
**Ovarium Follicle**	-	-	-	-	-
**HOMA IR**	0.828	-	-	-	-
**IGF1**	0.555	0.662	-	-	-
**PCOS**	0.929	0.858	0.565	-	-
**Zonulin**	0.686	0.63	0.431	0.717	-

**Table 8:** Blindfolding.

-	**SSO**	**SSE**	**Q^2^ (=1-SSE/SSO)**
**Ovarium Follicle**	40	28.557	0.286
**HOMA IR**	40	25.177	0.371
**IGF1**	40	23.426	0.414
**PCOS**	40	40	-
**Zonulin**	40	20.196	0.495

**Table 9:** Results of the coefficient of determination test.

**Variable**	**R Square**	**R Square Adjusted**	**Category**
**HOMA-IR**	0.397	0.382	Moderate
**IGF-1**	0.438	0.423	Moderate
**Zonulin**	0.714	0.511	Strong

**Table 10:** Q^2^ test.

**Variable**	**SSO**	**SSE**	**Q^2^ (=1-SSE/SSO)**
**HOMA- IR**	40.000	25.177	0.371
**IGF-1**	40.000	23.426	0.414
**Zonulin**	40.000	20.196	0.495

**Table 11:** Spearman correlation.

-	**No**	**PCOS**	***Lactobacillus *sp.**	**IGF1**	**Zonulin**	**HOMA IR**	**Ovarian Follicles**
**No**	1	-	-	-	-	-	-
**PCOS**	0.866	1	-	-	-	-	-
***Lactobacillus *sp.**	-0.732	-0.915	1	-	-	-	-
**IGF1**	-0.397	-0.565	0.518	1	-	-	-
**Zonulin**	-0.666	-0.717	0.644	0.431	1	-	-
**HOMA IR**	-0.696	-0.858	0.832	0.662	0.63	1	-
**Ovarian Follicles**	-0.808	-0.929	0.919	0.555	0.686	0.828	1

**Table 12:** Confidence interval.

-	**Original Sample (O)**	**Sample Mean (M)**	**2.50%**	**97.50%**
**HOMA IR → Ovarian Follicles**	0.37	0.37	0.17	0.56
**HOMA IR → IGF1**	0.66	0.66	0.48	0.8
**IGF1 → Ovarian Follicles**	0.56	0.55	0.35	0.71
**PCOS → Ovarian Follicles**	-0.17	-0.17	-0.3	-0.06
**PCOS →> HOMA IR**	-0.45	-0.46	-0.64	-0.27
**PCOS → IGF1**	-0.3	-0.3	-0.47	-0.16
**PCOS → Zonulin**	-0.72	-0.72	-0.84	-0.56
**Zonulin → Ovarian Follicles**	0.23	0.24	0.1	0.38
**Zonulin → HOMA IR**	0.63	0.64	0.45	0.79
**Zonulin → IGF1**	0.42	0.42	0.25	0.57

**Table 13:** Results of the direct effect hypothesis test in the structural model.

**Path**	**Standard Deviation (STDEV)**	**T Statistics (|O/STDEV|)**	***P* Values**
**HOMA IR → IGF1**	0.080	8.280	0.000
**PCOS → Zonulin**	0.073	9.797	0.000
**Zonulin → HOMA IR**	0.088	7.149	0.000

**Table 14:** Results of the indirect effect hypothesis test in the structural model.

**Path**	**Standard Deviation (STDEV)**	**T Statistics (|O/STDEV|)**	***P* Values**
**PCOS → Zonulin → HOMA IR**	0.096	4.688	0.000
**Zonulin → HOMA IR → IGF1**	0.085	4.909	0.000
**PCOS → Zonulin → HOMA IR → IGF1**	0.081	3.682	0.000

## Data Availability

All data generated or analyzed during this study are included in this published article.

## LIST OF ABBREVIATIONS

BMI: Body Mass Index
GR: Glucocorticoid Receptor
HOMA-IR: Homeostasis Model Assessment of Insulin Resistance
IGF-1: Insulin-Like Growth Factor-1
IRI: Insulin Resistance Index
LPS: Lipopolysaccharides
PCOS: Polycystic Ovary Syndrome
SCFAs: Short Chain Fatty Acids
SEM: Structural Equation Modeling
SEM-PLS: Structural Equation Modeling–Partial Least Squares
TLR4: Toll-like Receptor 4
