# Supplementary material for: Mechanism of Gut Microbiota Dysbiosis in Insulin Resistance Polycystic Ovary Syndrome
Source: Endocr Metab Immune Disord Drug Targets. 2026 Apr 13;26:E18715303429583. doi: 10.2174/0118715303429583260303181915 (PMC13670188; doi:10.2174/0118715303429583260303181915)
Supplement: Supplementary file 1 [file EMIDDT-26-E18715303429583_SD1.pdf]

Supplementary Material

Mechanism of Gut Microbiota Dysbiosis in Insulin Resistance Polycystic Ovary Syndrome

Fatimah Usman[1,*], Masagus Irsan Saleh[2], Kemas Yusuf Effendi[3], Peby Maulina Lestari[4] and Widjiati Widjiati[5]

[1]Doctoral Program of Biomedic Science, Faculty of Medicine, Sriwijaya University, Palembang, Indonesia; [2]Department of Pharmacology, Sriwijaya University, Palembang, Indonesia; [3]Division of Reproductive Endocrinology and Infertility, Department of Obstetrics and Gynecology, Dr. Mohammad Hoesin General Hospital, Palembang, Indonesia; [4]Division of Maternal-Fetal Medicine, Department of Obstetrics and Gynecology, Dr. Mohammad Hoesin General Hospital, Palembang, Indonesia; [5]Department of Embryology, Faculty of Veterinary Medicine, University of Airlangga, Surabaya, Indonesia

Data Figure 1

Analisis Uji Bivarite Perbandingan Zonulin

Report						
Zonulin						
Kelompok	N	Minimum	Maximum	Median	Mean	Std. Deviation
Kontrol	20	.5	6.0	3.800	3.775	1.1956
Perlakuan	20	2.9	6.9	5.850	5.545	1.1632
Total	40	.5	6.9	4.750	4.660	1.4693

Tests of Normality							
		Kolmogorov-Smirnov[a]			Shapiro-Wilk		
	Kelompok	Statistic	df	Sig.	Statistic	df	Sig.
Zonulin	Kontrol	.115	20	.200*	.953	20	.413
	Perlakuan	.153	20	.200*	.890	20	.027
*. This is a lower bound of the true significance.							
a. Lilliefors Significance Correction							

Tabel 14. Uji Mann-Whitiney Zonulin.

**Kelompok**	**Rerata deret**
**Kontrol**	13,40
**Perlakuan**	27,60

Tabel 15. Uji Signifikansi Mann-Whitney Zonulin.

**Uji Statistik Zonulin**	
**Mann-Whitney U**	58,00
**Asymp Sig. (2-tailed)**	>0,0001

Data Figure 2

Analisis Uji Bivarite Perbandingan HOMA-IR

Report						
HOMA IR						
Kelompok	N	Minimum	Maximum	Median	Mean	Std. Deviation
Kontrol	20	35.65	103.49	78.3400	78.8725	19.34804
Perlakuan	20	68.29	240.83	136.1900	140.9760	42.29508
Total	40	35.65	240.83	102.4950	109.9243	45.19747

Tests of Normality							
		Kolmogorov-Smirnov[a]			Shapiro-Wilk		
	Kelompok	Statistic	df	Sig.	Statistic	df	Sig.
HOMA IR	Kontrol	.118	20	.200*	.939	20	.231
	Perlakuan	.197	20	.042	.922	20	.106
*. This is a lower bound of the true significance.							
a. Lilliefors Significance Correction							

Tabel 16. Uji-T HOMA-IR

**Kelompok**	**Rerata deret**	**Simpangan baku**
**Kontrol**	78,873	19,348
**Perlakuan**	140,976	42,295

Tabel 17. Signifikansi Uji-T HOMA-IR

**Independent Samples Test HOMA-IR**			
**F**	t	df	Sig (2-tailed)
**4,090**	-5,971	38	<0,0001

Data Figure 3

Analisis Uji Bivarite Perbandingan IGF-1

Report						
IGF-1						
Kelompok	N	Minimum	Maximum	Median	Mean	Std. Deviation
Kontrol	20	36.97	229.72	150.9150	141.9665	44.05268
Perlakuan	20	102.79	304.65	186.8650	189.5700	53.49800
Total	40	36.97	304.65	162.4700	165.7683	54.04456

Tests of Normality							
		Kolmogorov-Smirnov[a]			Shapiro-Wilk		
	Kelompok	Statistic	df	Sig.	Statistic	df	Sig.
IGF1	Kontrol	.130	20	.200*	.977	20	.895
	Perlakuan	.128	20	.200*	.962	20	.589
*. This is a lower bound of the true significance.							
a. Lilliefors Significance Correction							

Tabel 18. Uji-t IGFI.

**Kelompok**	**Rerata deret**	**Simpangan baku**
**Kontrol**	141,967	44,053
**Perlakuan**	189,670	53,498

Tabel 19. Signifikansi Uji-T IGF-1.

**Independent Samples Test IGF-1**			
**F**	t	df	Sig (2-tailed)
**0,670**	-3,072	38	0,004

Data Figure 4

Path coefficients

	Original sample (O)	T statistics (|O/STDEV|)	P values
Bakteri_Lactobacillus sp -> ScFA	0.617	6.386	0.000
Bakteri_Lactobacillus sp -> Zonulin	-0.558	5.439	0.000
HOMA IR -> IGF1	0.339	2.699	0.007
IGF1 -> Folikel_Ovarium	-0.479	4.431	0.000
SOPK -> Bakteri_Lactobacillus sp	-0.915	26.049	0.000
ScFA -> IGF1	-0.419	3.325	0.001
Zonulin -> HOMA IR	0.448	3.150	0.002
